# Supplementary material for: Unsupervised identification of disease states from high‐dimensional physiological and histopathological profiles
Source: Mol Syst Biol. 2019 Feb 19;15(2):e8636. doi: 10.15252/msb.20188636 (PMC6380462; doi:10.15252/msb.20188636)
Supplement: Supplementary file 1 — Appendix [file MSB-15-e8636-s001.pdf]

APPENDIX.

Title: Unsupervised identification of disease states from high dimensional physiological and histopathological profiles

Running title: Machine identification of disease states

Authors: Kenichi Shimada[1,*], Timothy J. Mitchison[1]

Table of contents:

Appendix Figure S1 – Page 1

Appendix Figure S2 – Page 2 - 4

Appendix Figure S3 – Page 5 - 8

Appendix Figure S4 – Page 9

Appendix Figure S5 – Page 10

Appendix Figure S6 – Page 11

Appendix Figure S7 – Page 12

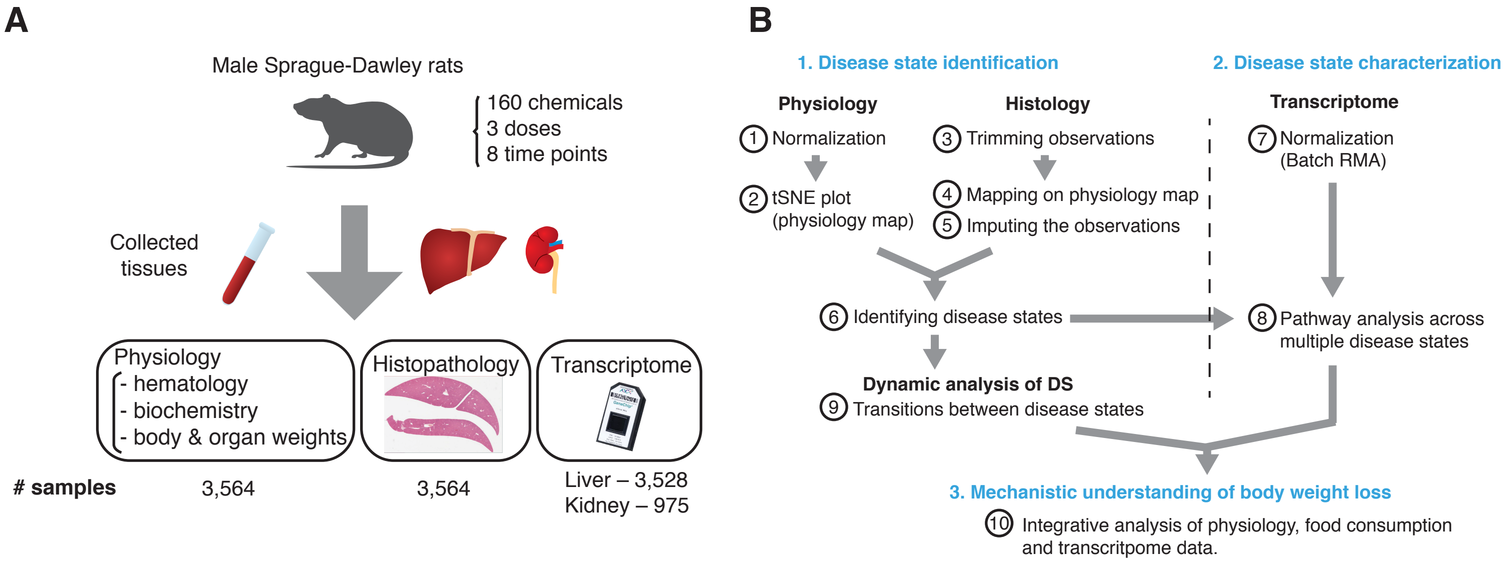

C

Category	Class	Subclass	Target	Structure
Drugs (99)	CNS (27)	anticonvulsant (9)	GABAA agonist (5)	benzodiazepine (2)
		antidipressant (8)	serotonin inh (6)	SNRI, tricyclics (4)
		antipsychotic (6)	D2 inh (6)	phenothiazine (4)
	metabolic disease (25)	diabetes (6)	SUR1 inh (3)	sulfonylurea (3)
		lipid-lowering (6)	PPARA agonist (5)	fibrate (4)
		gastric secretion inh (4)	H2 inh (4)	
		uricosuric (3)	XO inh (3)	
		diuretic (3)		
	antiinflammatory, COXs (19)	NSAID (13)	COXs inh (11)	acetic acid (4)
				oxicam (3)
				propionic acid (2)
			COX2 inh (2)	
	circulatory (17)	antiarrythmic (7)	VNaCh inh (5)	alkaloid (2)
		hypotensive (4)	ACE inh (2)	
		vasodilator (4)	CaCh inh (4)	
		antianginal (3)		
		antispasmodic (2)		
	antiinflammatory, allergy (14)	antihistamine (14)	H1 inh (7)	
			H2 inh (4)	
		anti-rheumatic (4)		
	hormone (8)	androgen (2)	AR	
		antithyroid (2)	thyroid peroxidase inh (2)	
		estrogen (2)	ER	
		muscle relaxant (2)		
	PNS (2)			
Toxins (55)	toxin (27)	livertox (9)	DNA inh (4)	nitroso compound (4)
				nitroso compound, nitrosourea (2)
		alcohol (2)		
	antimicrobial (19)	antibacterial (14)		
		antibiotic (8)		
		antifungal (5)	fungal ergosterol synthesis inh (2)	
		tubercurosis (4)		
		synthetic antibacterial (2)		
	anticancer (13)		DNA inh (3)	containing Pt (2)
Endogenous (6)	endogenous (6)	vitamin (2)		

Appendix Figure S1. Experimental and analytical workflow in this study

A Experimental flow in Open TG-GATEs.

B Analytical workflow in this study.

C Known biological activity of 160 compounds investigated *in vivo* in Open TG-GATEs. Only the features shared by more than one compound are shown.

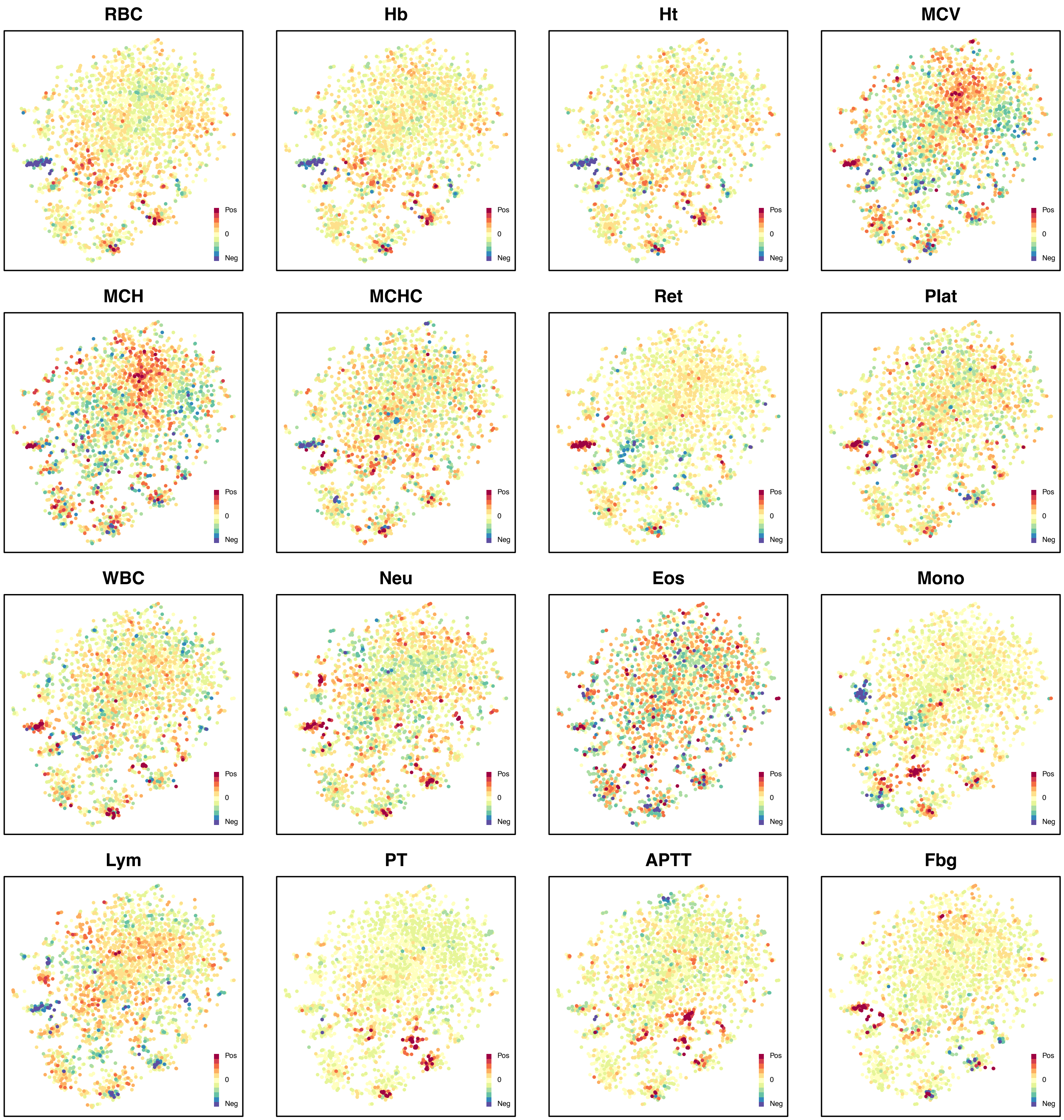

Appendix Figure S2. Physiology parameters on t-SNE map (1/3 page)
46 physiology parameters, as well as time point and relative doses (Low, Middle, High) of the measurements were mapped onto the physiology t-SNE map.

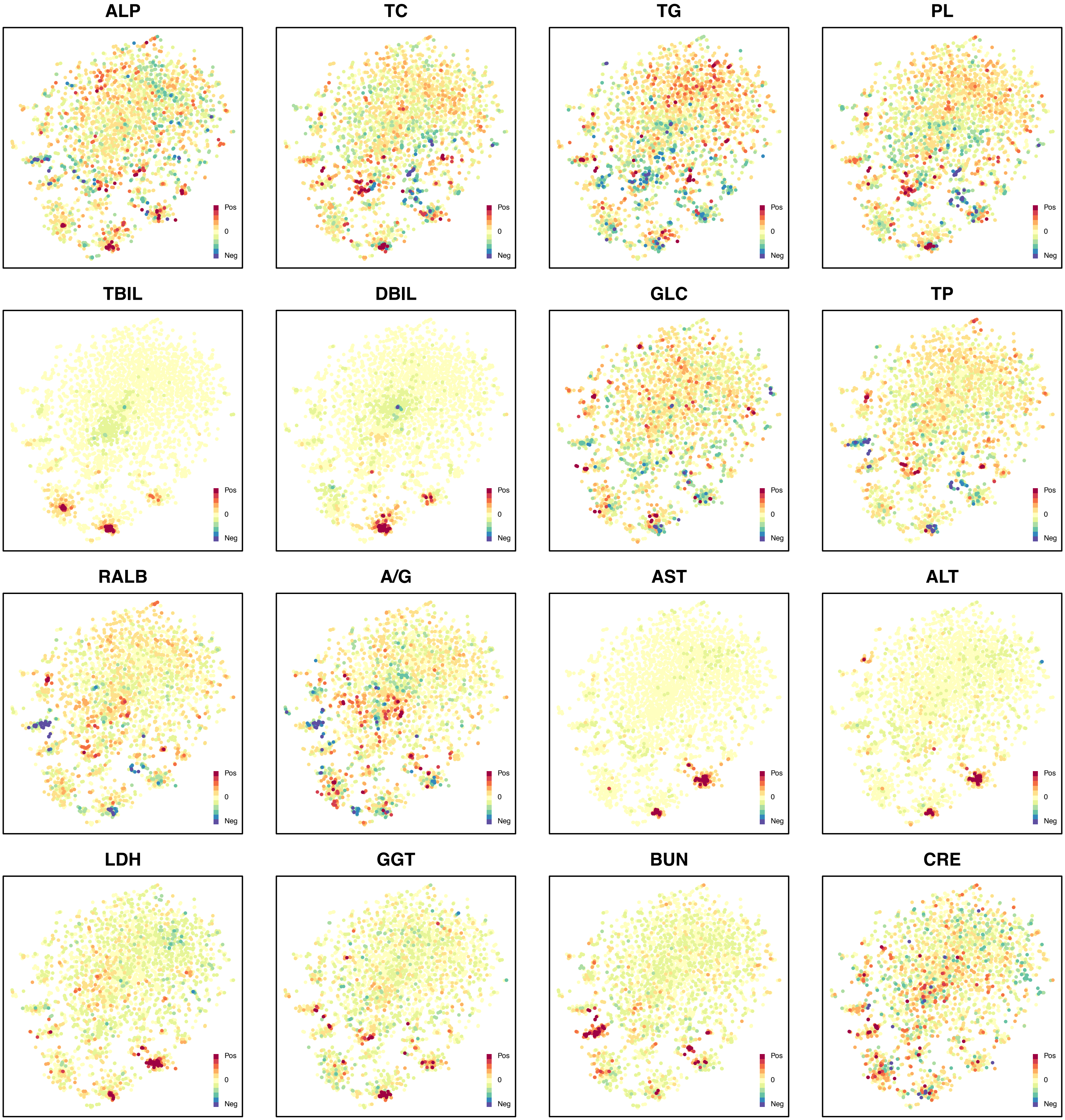

Appendix Figure S2. Physiology parameters on t-SNE map (continued, 2/3 page)
46 physiology parameters, as well as time point and relative doses (Low, Middle, High) of the measurements were mapped onto the physiology t-SNE map.

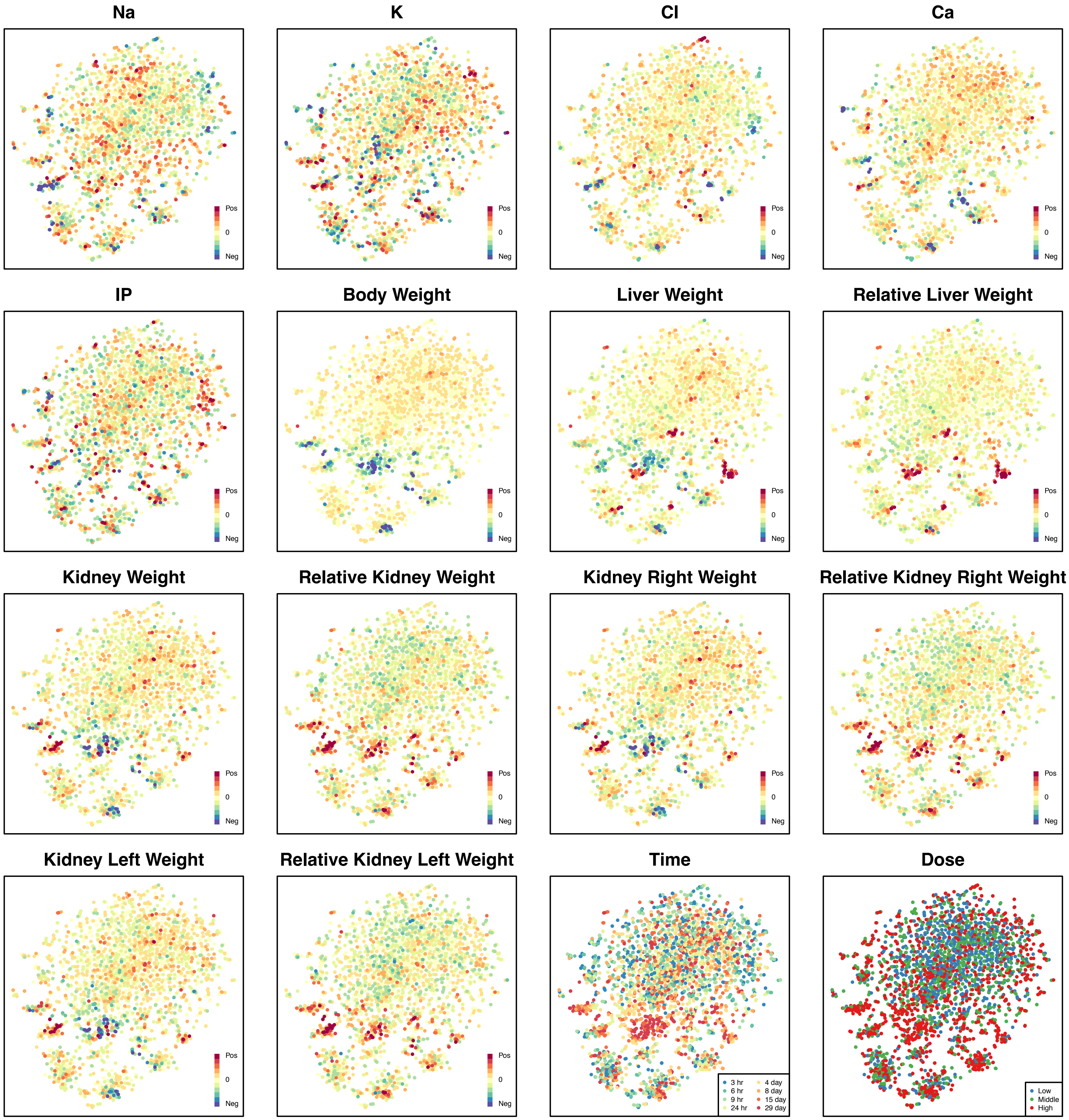

Appendix Figure S2. Physiology parameters on t-SNE map (continued, 3/3 page)
46 physiology parameters, as well as time point and relative doses (Low, Middle, High) of the measurements were mapped onto the physiology t-SNE map.

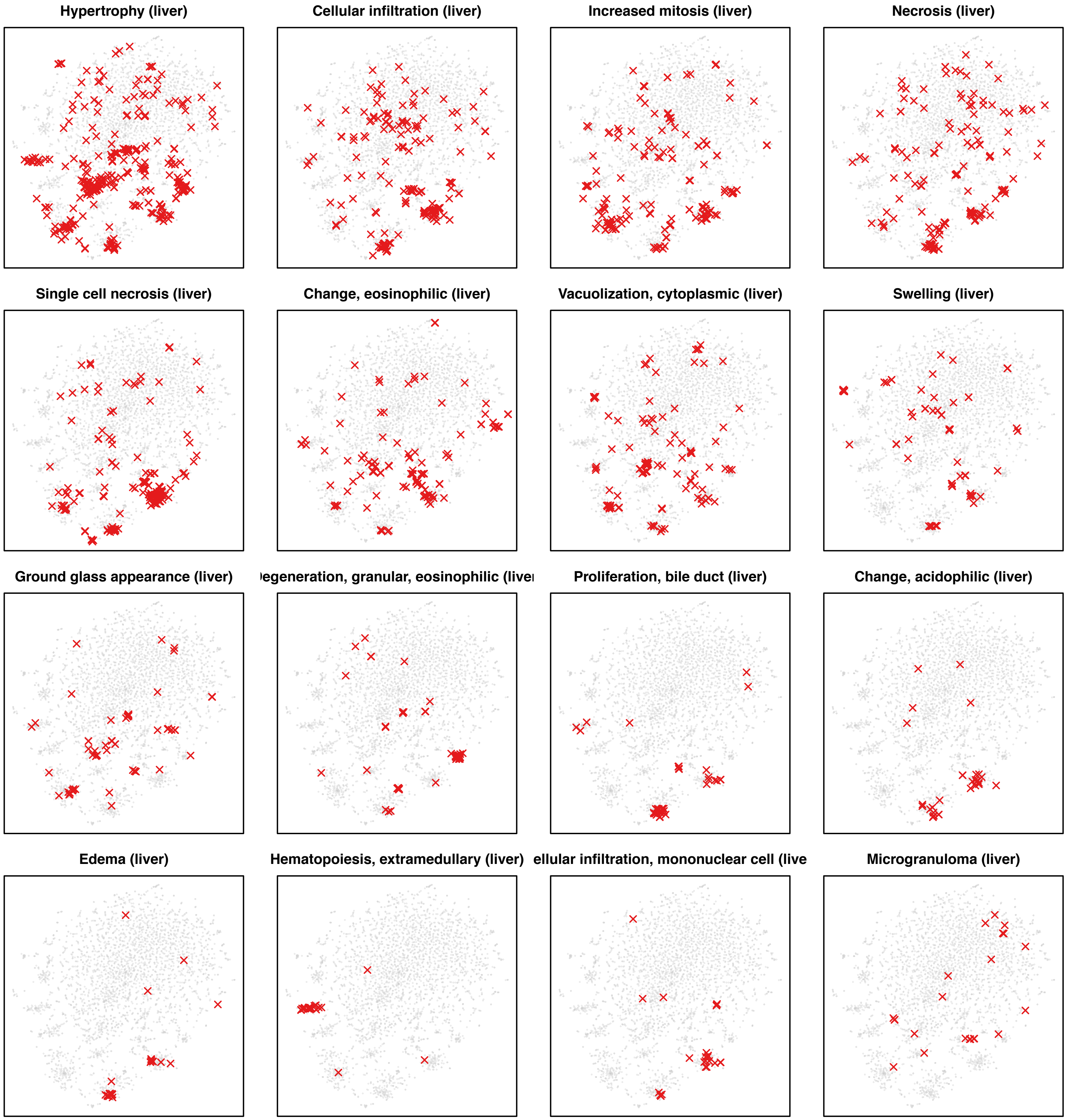

Appendix Figure S3. Histopathology mapped onto the physiology t-SNE (1/4 page)
Observations of each histopathology phenotypes in the liver (red) and kidney (blue) were mapped onto the physiology t-SNE plot.

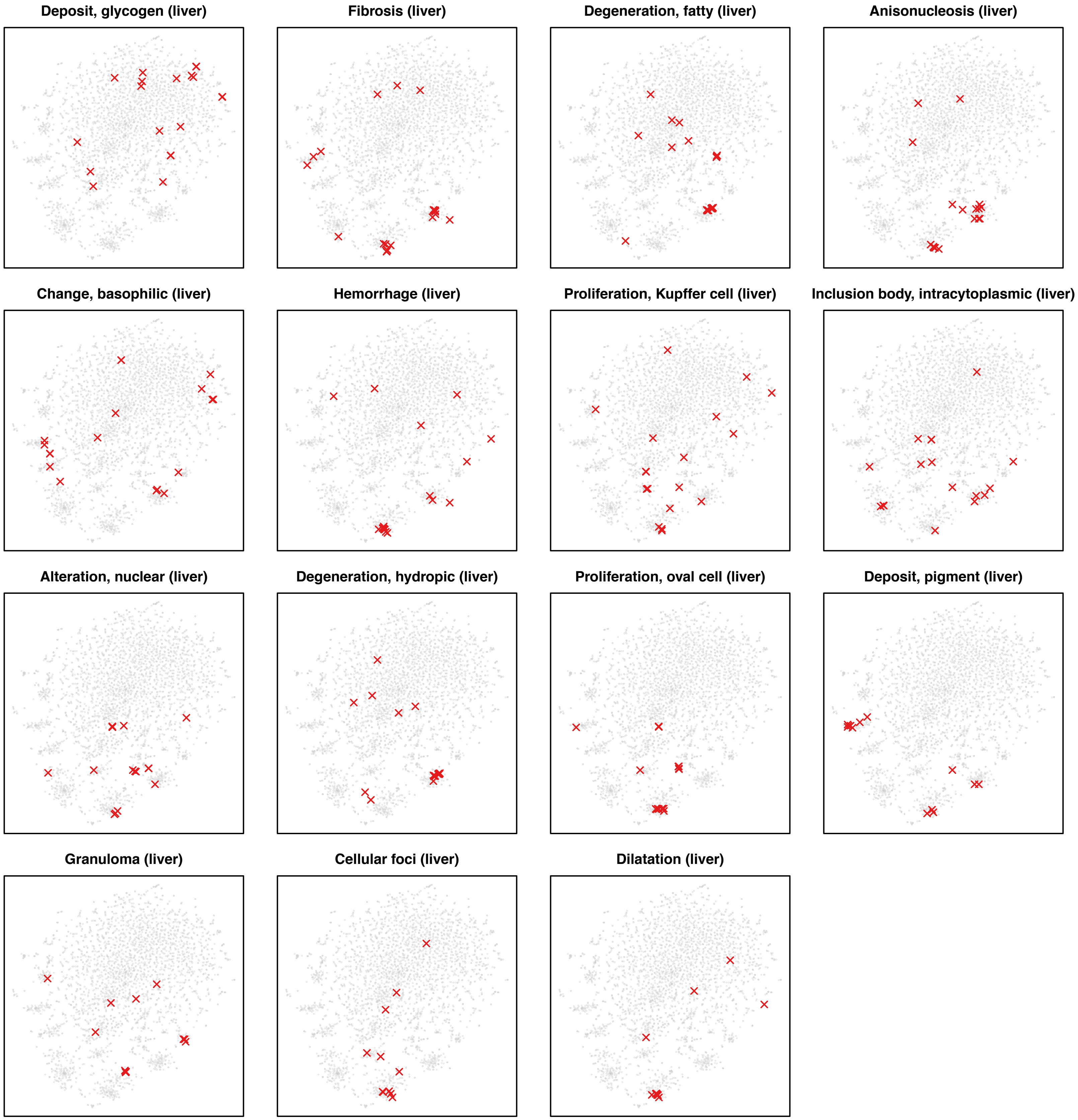

Appendix Figure S3. Histopathology mapped onto the physiology t-SNE (cont'd, 2/4 page)
Observations of each histopathology phenotypes in the liver (red) and kidney (blue) were mapped onto the physiology t-SNE plot.

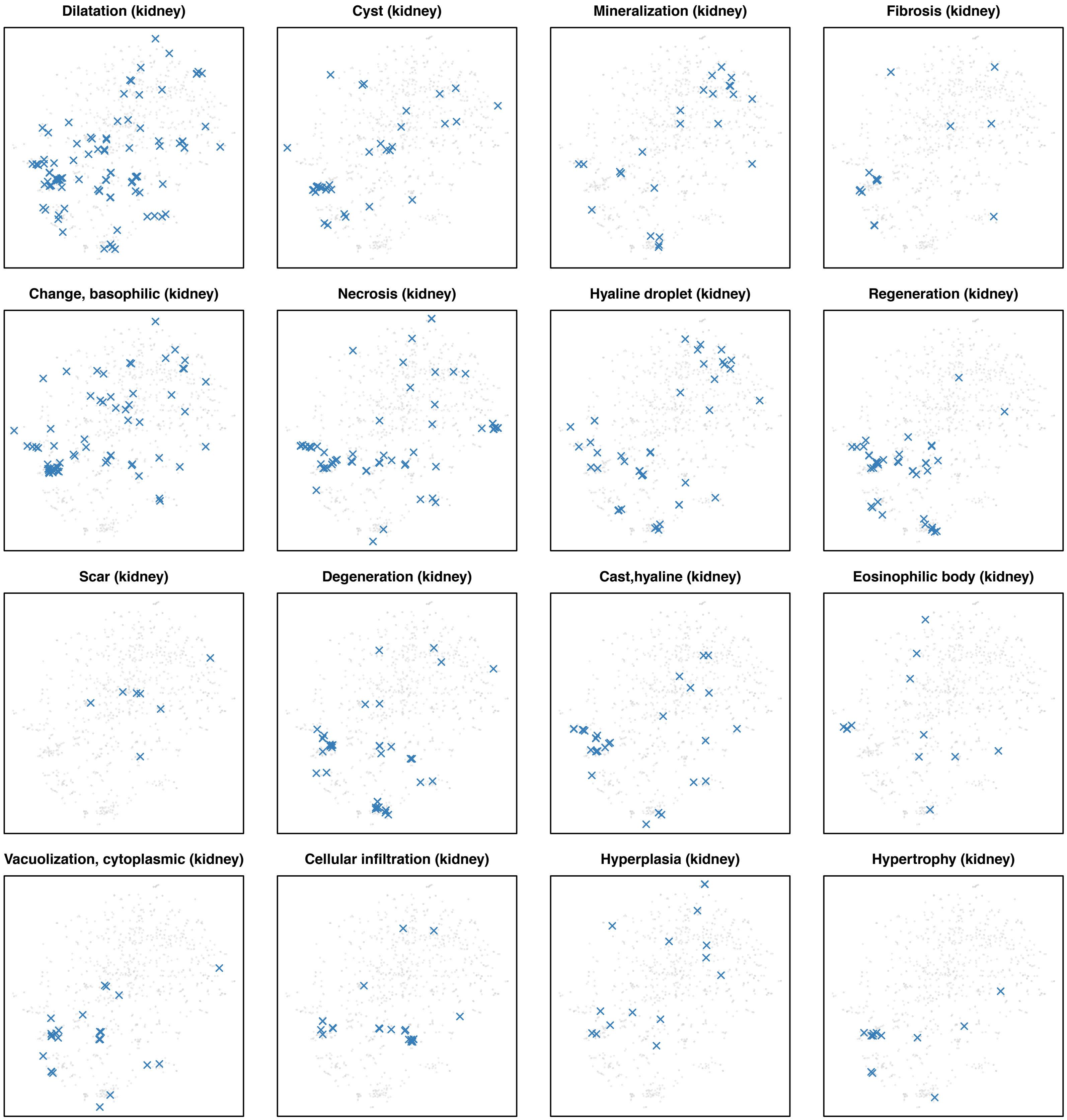

Appendix Figure S3. Histopathology mapped onto the physiology t-SNE (cont'd, 3/4 page)
Observations of each histopathology phenotypes in the liver (red) and kidney (blue) were mapped onto the physiology t-SNE plot.

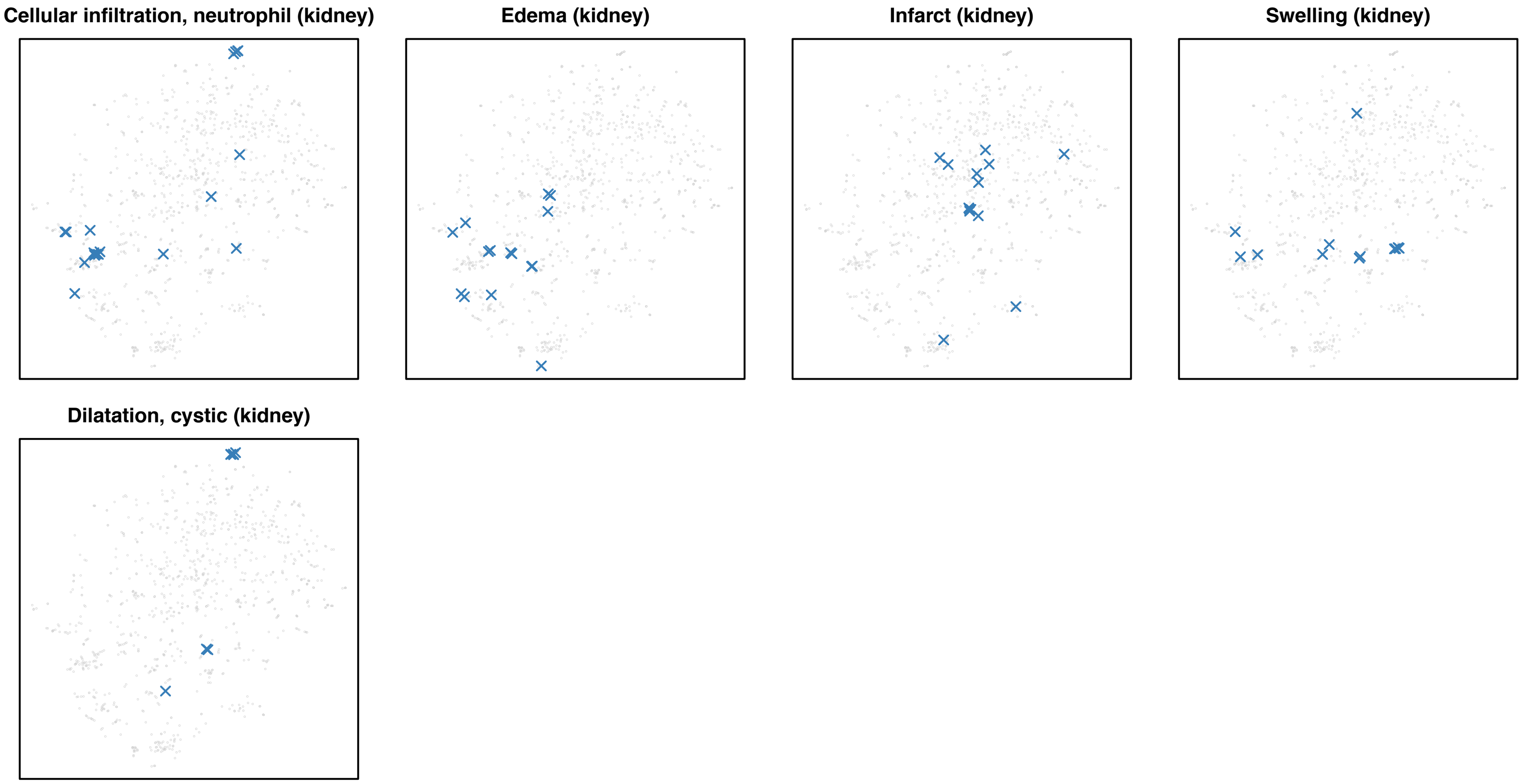

Appendix Figure S3. Histopathology mapped onto the physiology t-SNE (cont'd, 4/4 page)
Observations of each histopathology phenotypes in the liver (red) and kidney (blue) were mapped onto the physiology t-SNE plot.

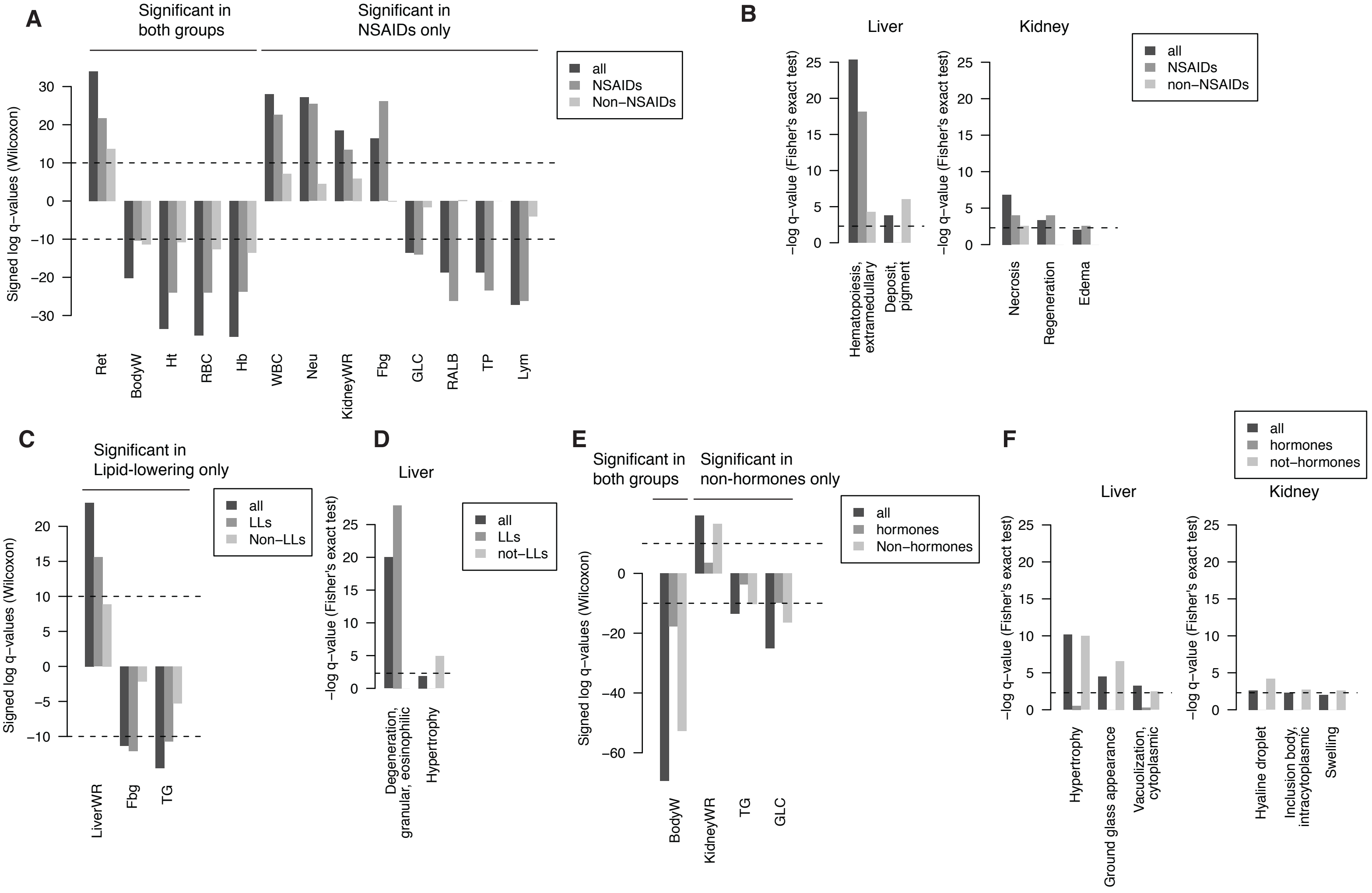

Appendix Figure S4. Toxin class overrepresentations in DS

A Changes in physiological parameters among DS8 that are NSAIDs and not.

B Overrepresented histopathological phenotypes among DS8 that are NSAIDs and not.

C Changes in physiological parameters among DS1 that are lipid-lowering compounds (LLs) or not.

D Overrepresented histopathological phenotypes among DS1 that are LLs or not.

E Physiological changes of hormone and non-hormone DS2.

F Histopathological overrepresentation of hormone or non-hormone DS2.

For **A-F**, Thresholds for FDR-adjusted p-values (q-values) to call significant were set as $1x10^{-10}$ for physiology and $5x10^{-3}$ for histology.

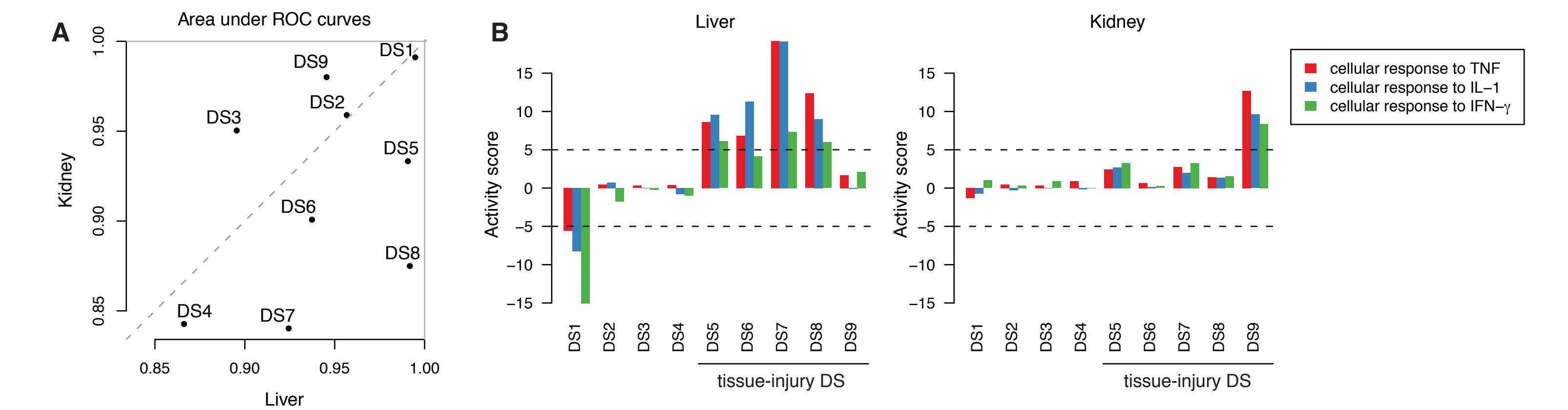

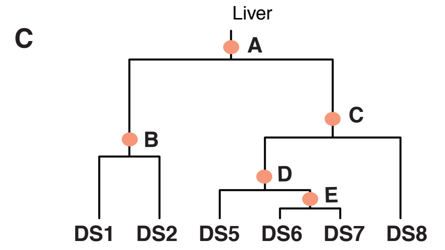

D

	A	B	C	D	E	DS1	DS2	DS5	DS6	DS7	DS8	Total
activated	2	3	13	25	15	45	14	15	2	29	112	275
suppressed	1	6	15	16	37	191	1	26	24	17	33	367

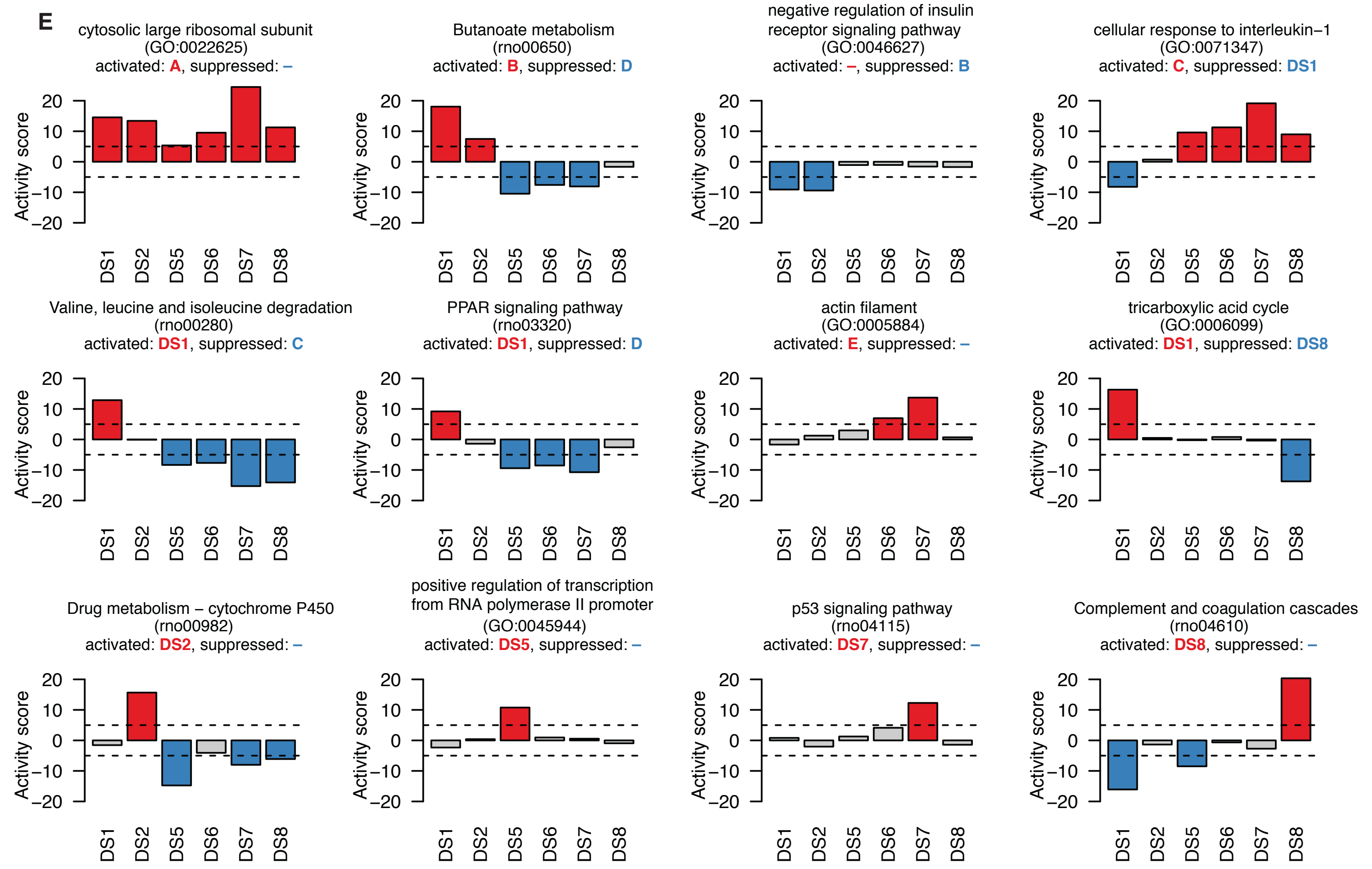

Appendix FIgure S5. Transcriptomic characterization of DS

A Area under ROC curves from elastic net classifiers for each DS using either liver or kidney transcriptome data.

B Transcriptional activity of GO terms ‘cellular responses to individual proinflammatory cytokines (TNF, IFN-γ, IL-1)’ against each DS.

C Dendrogram of the six DSs which showed substantial liver transcriptome changes. Nodes A-E corresponds to multiple DSs.

D Number of activated and suppressed pathways in each node (individual DS or multiple DSs).

E Examples of 12 pathways and the corresponding nodes in which they were activated or suppressed. In some cases, such as suppression of rno00982 or rno04610, pathways were not assigned to any nodes because the pattern of activation/suppression did not uniquely match to any nodes.

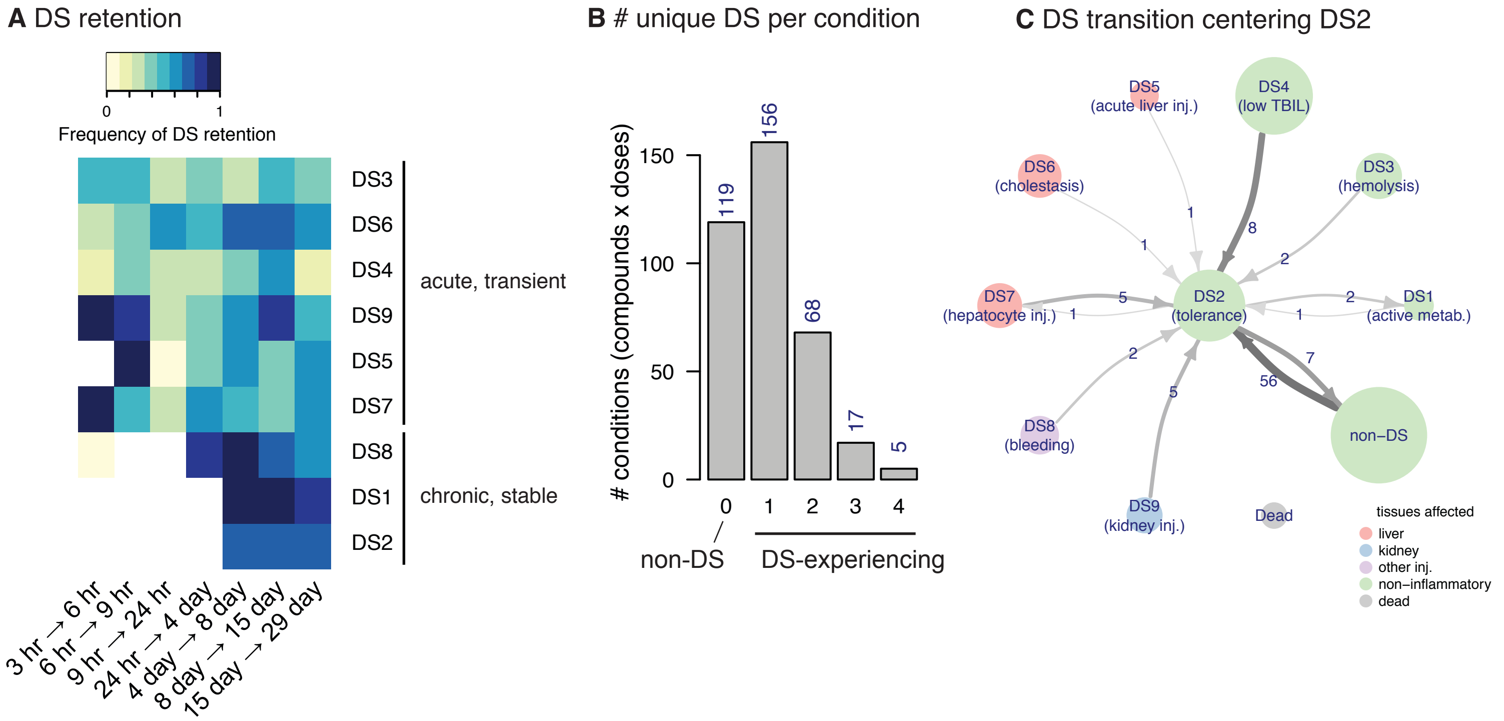

Appendix Figure S6. Disease state dynamics

A DS retention. A heatmap showing the ratio of conditions which retain the same DSs between two consecutive time points.

B The number of unique DSs caused by one condition is summarized.

C Same as DS transition graph in Fig. 4B except that this one highlights changes to and from DS2 only.

A

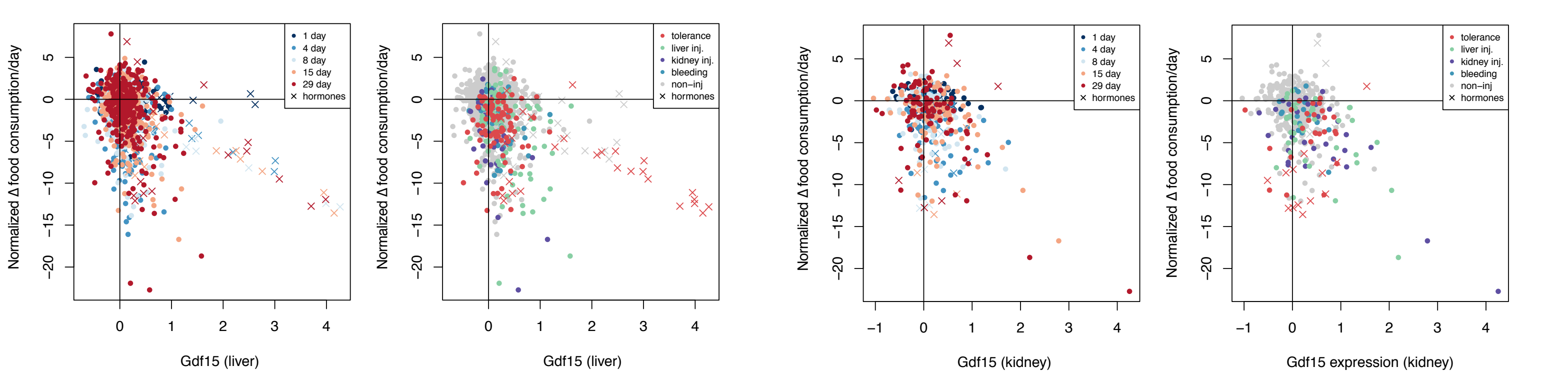

C

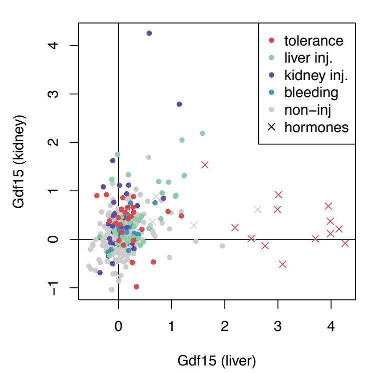

D Linear regression coefficients with symthetic hormone data

	tissue	Estimate	Std. Error	t value	Pr(>ltl)
All	liver	-2.31	0.22	-10.72	**3.72E-24**
	kidney	-2.28	0.29	-7.94	**1.51E-14**
tolerance	liver	-2.71	0.24	-11.52	**3.68E-27**
	kidney	-0.34	0.79	-0.43	6.64E-01
liver inj.	liver	-3.22	1.24	-2.60	9.55E-03
	kidney	-2.50	0.89	-2.81	5.16E-03
kidney inj.	liver	-4.73	1.91	-2.48	1.33E-02
	kidney	-3.77	0.57	-6.64	**8.68E-11**
bleeding	liver	-2.01	4.13	-0.49	6.27E-01
	kidney	-3.35	2.93	-1.14	2.53E-01
non injury	liver	-0.86	0.45	-1.90	5.86E-02
	kidney	-0.87	0.45	-1.95	5.19E-02

E Linear regression coefficients without symthetic hormone data

	tissue	Estimate	Std. Error	t value	Pr(>ltl)
All	liver	-2.31	0.47	-4.89	**1.38E-06**
	kidney	-2.59	0.30	-8.62	**1.17E-16**
tolerance	liver	-2.89	1.41	-2.04	4.20E-02
	kidney	-2.00	0.95	-2.10	3.66E-02
liver inj.	liver	-3.22	1.22	-2.63	8.87E-03
	kidney	-2.50	0.88	-2.84	4.75E-03
kidney inj.	liver	-4.73	1.89	-2.51	1.25E-02
	kidney	-3.77	0.56	-6.71	**6.21E-11**
bleeding	liver	-2.01	4.09	-0.49	6.23E-01
	kidney	-3.36	2.90	-1.16	2.48E-01
non injury	liver	-1.72	0.57	-3.00	2.89E-03
	kidney	-0.96	0.45	-2.10	3.60E-02

Appendix Figure S7. Relationship between Gdf15 transcription and food consumption

A-B Liver **(A)** or kidney **(B)** expression of Gdf15 and food consumption was plotted. Points were color coded by time points (left) and disease states (right). Conditions treated with synthetic hormones were marked by x (otherwise filled circles).

C Gdf15's expression in liver and kidney are plotted. Points are color coded by disease states. Synthetic hormones are marked by x.

D-E Summary of multivariate linear regresion of food consumption on liver and kidney Gdf15 expressions. Regression was computed using data including synthetic hormone treatments **(D)**, or excluding them **(E)**. In both, p-values lower than 10^{-5} were highlighted in bold letters.
