# Supplementary material for: Unsupervised identification of disease states from high‐dimensional physiological and histopathological profiles
Source: Mol Syst Biol. 2019 Feb 19;15(2):e8636. doi: 10.15252/msb.20188636 (PMC6380462; doi:10.15252/msb.20188636)
Supplement: Supplementary file 2 — Expanded View Figures PDF [file MSB-15-e8636-s002.pdf]

Expanded View Figures

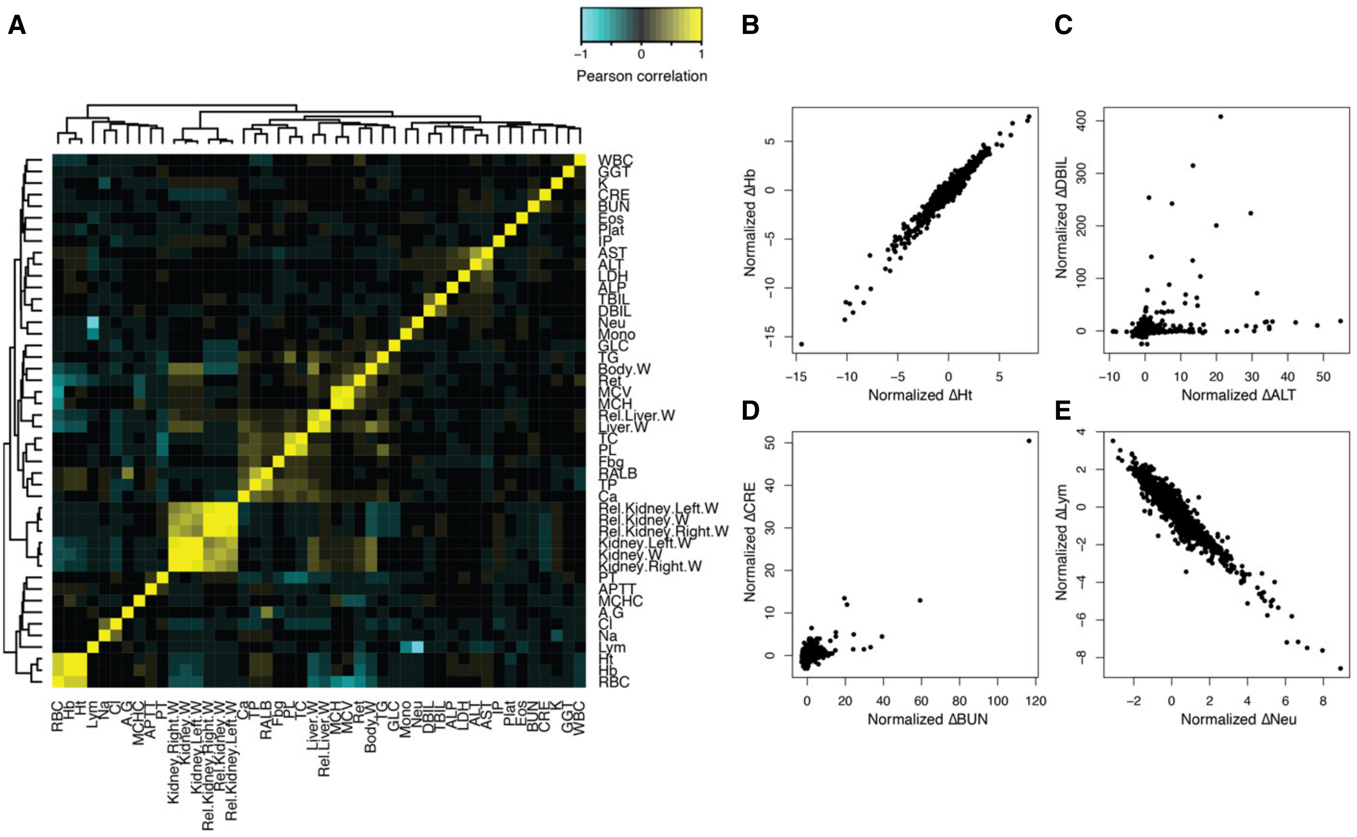

Figure EV1. Physiological parameters and their correlations.

A Heatmap showing Spearman correlation coefficients between physiological parameters across 3,564 conditions.

B–E Scatter plots of four parameter pairs across 3,564 samples. Note that each parameter of each treatment was normalized by subtracting control vehicle treatments and divided by interquartile range IQR across all 3,564 conditions. (B) Example of strong positive correlation: Ht and Hb. (C) Comparison of two liver injury markers: ALT and DBIL. (D) Comparison of two kidney injury markers: BUN and CRE. (E) Example of strong negative correlation: Neu and Lym.

 © 2019 The Authors

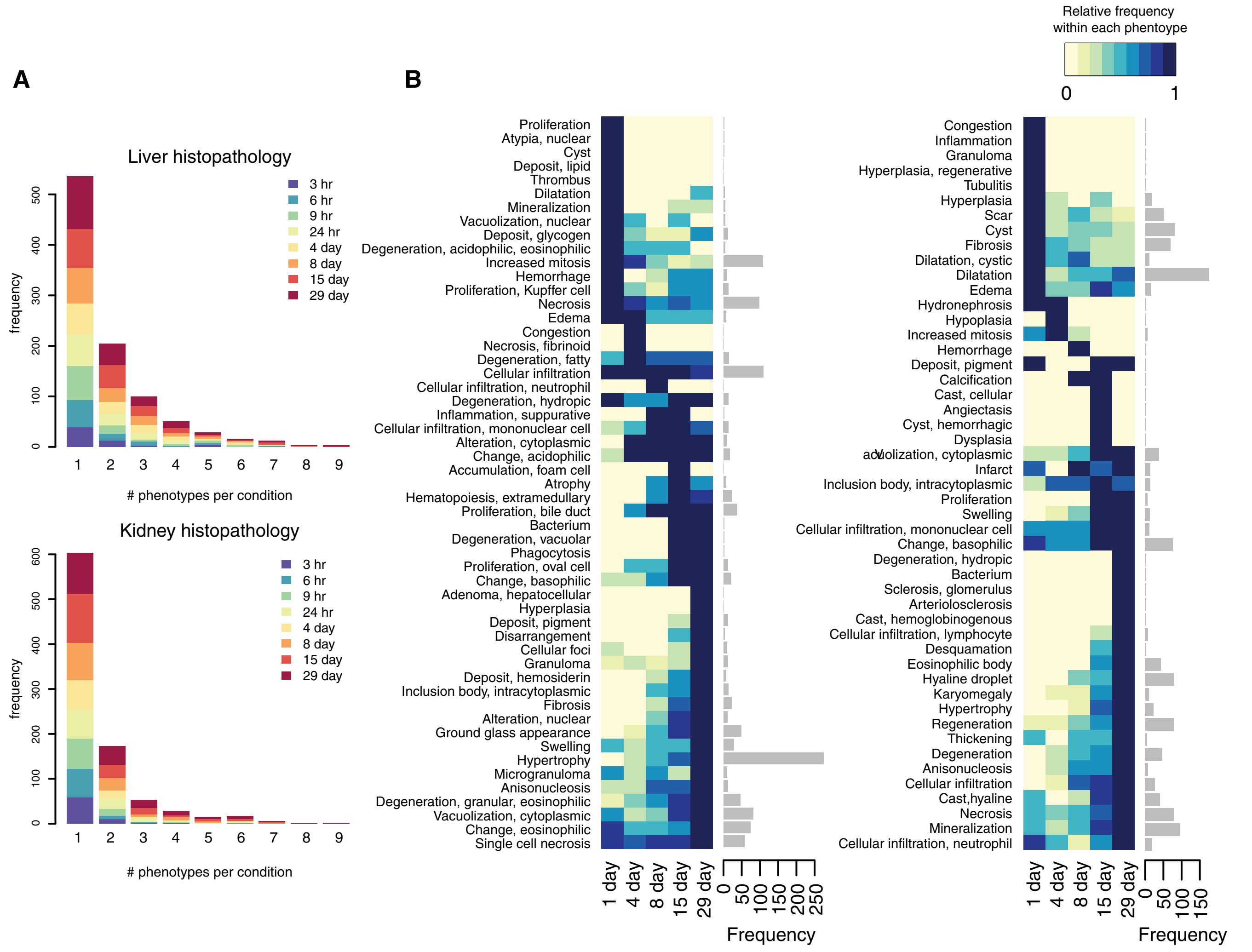

Figure EV2. Kinetics of histopathology phenotypes.

A Number of histopathology phenotypes observed per condition, stratified by time points.

B Kinetics of each histopathology phenotype. Color codes on the heatmap show relative frequency within each phenotype (i.e., normalized row-wise). Barplots on the right indicate the number of observations per phenotype.

© 2019 The Authors

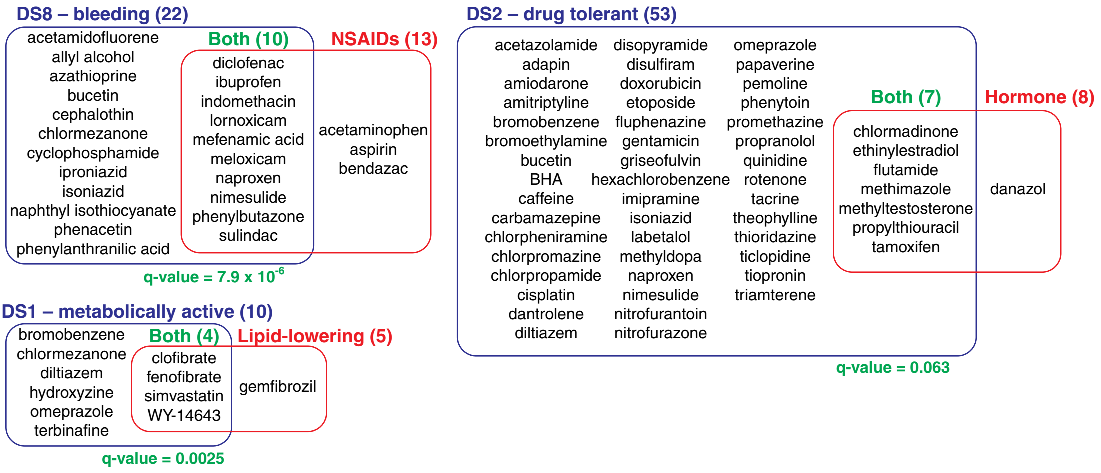

Figure EV3. Toxin class overrepresentations in DSs.

Compound classes overrepresented in three DSs (q-value $< 10^{-2}$).

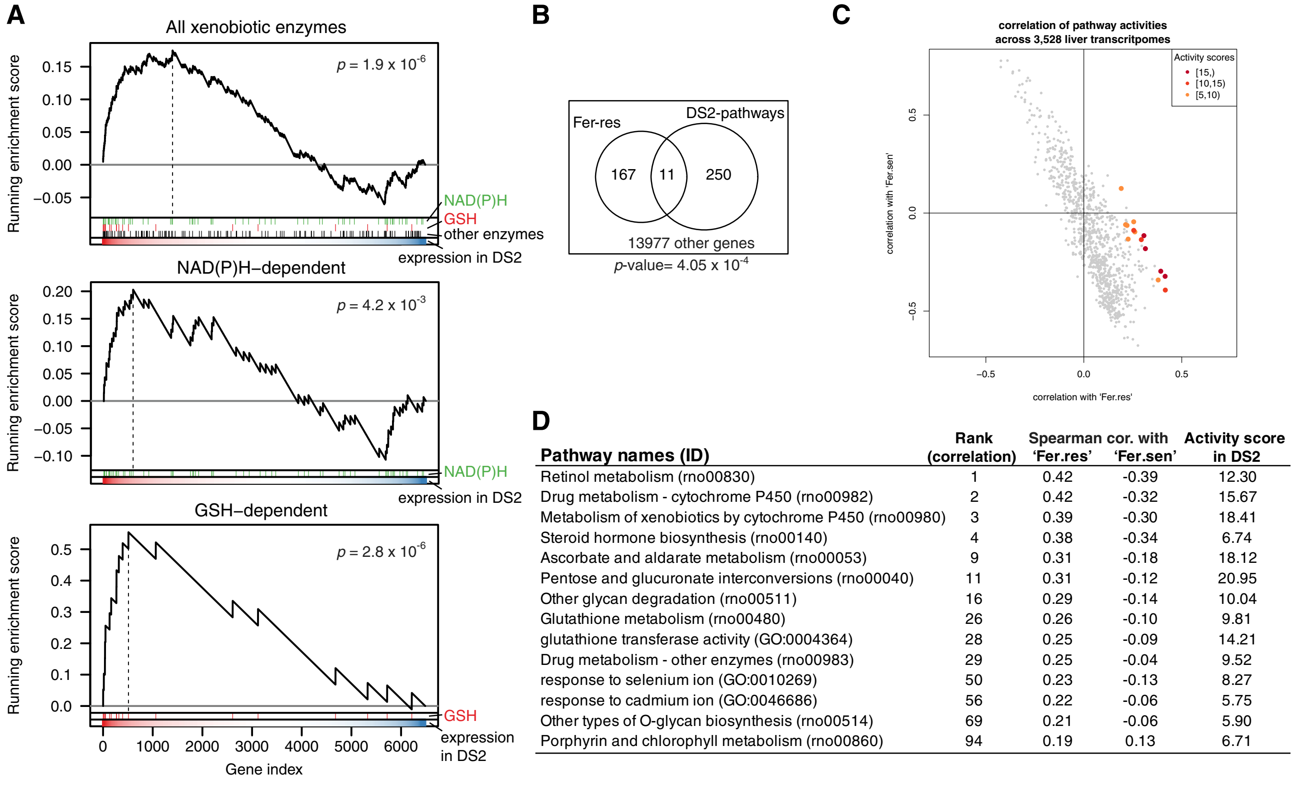

Pathway names (ID)	Rank (correlation)	Spearman cor. with 'Fer.res'	Spearman cor. with 'Fer.sen'	Activity score in DS2
Retinol metabolism (rno00830)	1	0.42	-0.39	12.30
Drug metabolism - cytochrome P450 (rno00982)	2	0.42	-0.32	15.67
Metabolism of xenobiotics by cytochrome P450 (rno00980)	3	0.39	-0.30	18.41
Steroid hormone biosynthesis (rno00140)	4	0.38	-0.34	6.74
Ascorbate and aldarate metabolism (rno00053)	9	0.31	-0.18	18.12
Pentose and glucuronate interconversions (rno00040)	11	0.31	-0.12	20.95
Other glycan degradation (rno00511)	16	0.29	-0.14	10.04
Glutathione metabolism (rno00480)	26	0.26	-0.10	9.81
glutathione transferase activity (GO:0004364)	28	0.25	-0.09	14.21
Drug metabolism - other enzymes (rno00983)	29	0.25	-0.04	9.52
response to selenium ion (GO:0010269)	50	0.23	-0.13	8.27
response to cadmium ion (GO:0046686)	56	0.22	-0.06	5.75
Other types of O-glycan biosynthesis (rno00514)	69	0.21	-0.06	5.90
Porphyrin and chlorophyll metabolism (rno00860)	94	0.19	0.13	6.71

Figure EV4. Induced drug tolerance is partly due to resistance to ferroptosis.

A GSEA of xenobiotic enzymes in DS2. Enrichment of genes encoding all xenobiotic metabolizing enzymes (top) as well as NAD(P)H (middle) and GSH (bottom) utilizing ones in DS2.

B Overlap between genes in Fer-res signature and the 14 pathways exclusively upregulated in DS2. P-value was calculated with one-sided Fisher's exact test.

C Spearman correlation coefficients of pathway activities between Fer-sen/Fer-res and 914 GO and KEGG pathways. Red points correspond to the 14 pathways specifically upregulated in DS2 and their activity scores in DS2.

D Spearman correlation coefficients of the 14 DS2-specifically upregulated pathways against Fer-sen and Fer-res.

 © 2019 The Authors

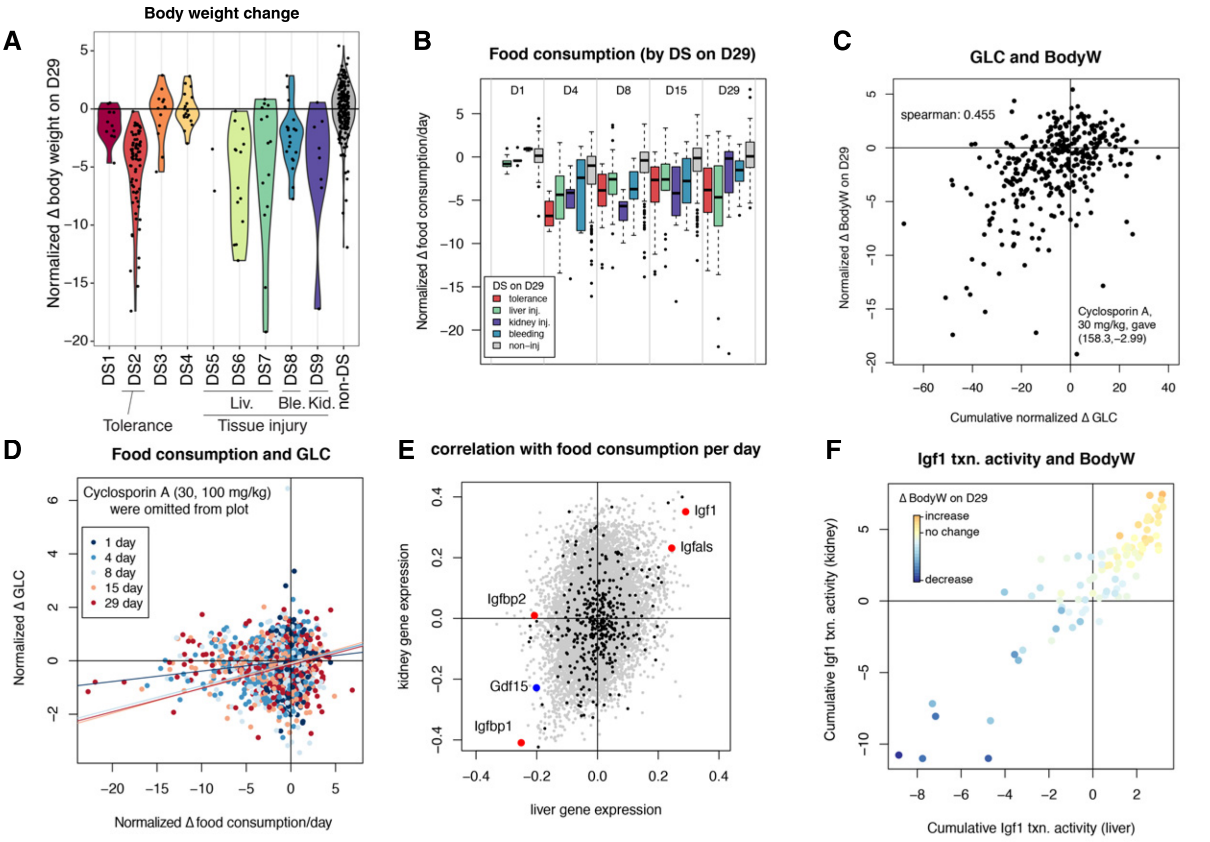

G

with synthetic hormones:

		Estimate	Std.err	z-value	P(>lzl)
Liver	Igf1	1.000			
	Igfals	1.785	0.162	11.045	0.000
	Igfbp1	-4.315	0.512	-0.842	0.000
	Igfbp2	-3.164	0.342	-9.240	0.000
Kidney	Igf1	1.000			
	Igfals	0.714	0.232	3.070	0.002
	Igfbp1	-7.088	3.518	-2.015	0.044
	Igfbp2	0.281	0.113	2.486	0.013

without synthetic hormones:

		Estimate	Std.err	z-value	P(>lzl)
Liver	Igf1	1.000			
	Igfals	2.202	0.210	10.498	0.000
	Igfbp1	-4.123	0.684	-6.024	0.000
	Igfbp2	-4.196	0.452	-9.276	0.000
Kidney	Igf1	1.000			
	Igfals	1.023	0.217	4.725	0.000
	Igfbp1	-2.357	0.449	-5.252	0.000
	Igfbp2	-0.305	0.112	-2.726	0.006

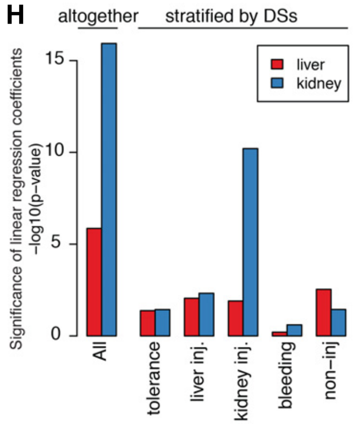

Figure EV5. Toxin-induced body weight loss mediated by Igf1 and Gdf15.

A Changes in normalized body weight on Day 29. The violin plot shows continuous distribution of the normalized body weight for each DS. Each distribution is scaled to have the same maximum width.

B Food consumption data were stratified by disease states at five time points. Note that tolerance (DS2) was observed on Day 4 at earliest. The horizontal lines, box limits and whiskers of the boxplot indicates the median, lower and upper quartiles and the most extreme data point which is no more than 1.5 times the interquartile range from the box.

C Relationship between cumulative blood glucose level and body weight on Day 29. Cyclosporine A at 30 mg/kg inducing hyperglycemia continuously therefore became an outlier.

D Relationship between food consumption and blood glucose level. A linear regression line for each time point was overlaid. Cyclosporine A treatments were omitted from the plots because they gave high GLC outliers, but they were used for linear regression.

E Spearman correlations of each gene's expression with food consumption at five time points (1, 4, 8, 15, and 29 days) among 337 and 95 conditions (compounds and doses) for liver and kidney, respectively.

F Cumulative Igf1 activities in liver and kidney using latent variable modeling. The plot is the same with Fig 6F, except for excluding synthetic hormone treatments (6/91 treatments). Points were color-coded by body weight change on Day 29.

G Contributions of the Igf1 system genes in the latent variable regression of body weight (Fig 6E), with (left) or without (right) synthetic hormones, respectively.

H Significance of contributions of liver and kidney Gdf15 expressions in linear regression of food consumption, with or without stratification by disease states.

© 2019 The Authors
